# Atomic Force Microscopy Imaging of Elastin Nanofibers Self-Assembly

**DOI:** 10.3390/ma16124313

**Published:** 2023-06-11

**Authors:** Kyriaki Sambani, Stylianos Vasileios Kontomaris, Dido Yova

**Affiliations:** 1 Biomedical Optics and Applied Biophysics Laboratory, Division of Electromagnetics, School of Electrical and Computer Engineering, Electrooptics and Electronic Materials, National Technical University of Athens, 9, Iroon Polytechniou, 15780 Athens, Greece; didoy@central.ntua.gr; 2 Faculty of Engineering and Architecture, Metropolitan College, 15125 Athens, Greece; 3 BioNanoTec Ltd., 2404 Nicosia, Cyprus

**Keywords:** elastin fibrils, atomic force microscopy, nanoscale imaging, self-assembly, nanobiomaterials, tissue engineering

## Abstract

Elastin is an extracellular matrix protein, providing elasticity to the organs, such as skin, blood vessels, lungs and elastic ligaments, presenting self-assembling ability to form elastic fibers. The elastin protein, as a component of elastin fibers, is one of the major proteins found in connective tissue and is responsible for the elasticity of tissues. It provides resilience to the human body, assembled as a continuous mesh of fibers that require to be deformed repetitively and reversibly. Thus, it is of great importance to investigate the development of the nanostructural surface of elastin-based biomaterials. The purpose of this research was to image the self-assembling process of elastin fiber structure under different experimental parameters such as suspension medium, elastin concentration, temperature of stock suspension and time interval after the preparation of the stock suspension. atomic force microscopy (AFM) was applied in order to investigate how different experimental parameters affected fiber development and morphology. The results demonstrated that through altering a number of experimental parameters, it was possible to affect the self-assembly procedure of elastin fibers from nanofibers and the formation of elastin nanostructured mesh consisting of naturally occurring fibers. Further clarification of the contribution of different parameters on fibril formation will enable the design and control of elastin-based nanobiomaterials with predetermined characteristics.

## 1. Introduction

Tissue engineering is an outgrowth of the biomaterials field, which involves the development of tissue substitutes to create target tissue whose structural characteristics and function need to be restored. The extracellular matrix (ECM) component of tissues is a complex structure surrounding and supporting the tissues. It is composed of major classes of biomolecules, such as elastin. The natural ECM has been a source of inspiration for the design and the production of biomaterials of potential interest in tissue engineering [1,2]. Elastin is an ECM protein responsible for the elastic properties of organs and tissues. As a result, it is of great importance in the perspective of producing biomaterials of potential interest in the nanotechnology and biotechnology fields [3]. 

One of the families of protein polymers obtained through genetic engineering attracting growing attention is elastin-like polypeptides (ELPs). Kowalczyk et al. [4] have analyzed selected applications of ELPs in medicine, therapeutic peptide delivery, drug delivery and tissue engineering. Creating biomaterials made of ELPs means that tissue engineering is achieved via several methods, such as creating coacervates and cross-linking [5,6,7]. 

The development of advanced biomaterials is often inspired by biological self-assembling modules, where simple building blocks are able to form complex natural systems. Bochicchio et al. [8] have demonstrated the self-assembly of a peptide in aqueous solution into elastin-like fibers at room temperature, examined using TEM. Elastin produces well-defined fibrils and fibers with specific mechanical and supramolecular properties [9]. Among proteins which are able to self-assemble, elastin has characteristics with repetitive sequences of small size and complexity responsible for self-assembly as well as for its elastic properties [10].

Self-assembly, known as a ‘bottom-up’ method, yields fibers with small diameters, and it offers novel properties and functionalities which cannot be achieved through conventional organic synthesis. Self-assembly of fibers refers to the build-up of nanoscale fibers from smaller molecules. The main disadvantage of the method is that it is an extremely complex and time-consuming technique. The ELP aggregation mechanism, modified by Rodriguez-Cabello et al. [11], resulted in fibrils that had a width in the nanometer range, which then increased to the micrometer range after aggregation. The coacervation process is reminiscent of elastin-derived peptides where the spontaneous self-assembly of monomers is induced via a temperature increase depending on protein and salt concentration [12]. Analysis of fibers via transmission electron microscopy revealed bundles of linear fibers in the nanometer range reflecting the common filamentous nature of elastomeric proteins [13].

The complexity of elastic fibers, combined with the unique physical properties of its component proteins, has made the understanding of elastin assembly process one of the most difficult problems. Although the structure of elastin has not been fully investigated, the way that structure relates to its properties is an area of intensive research. In this paper, the self-assembly process of elastin fibers under different conditions including suspension medium, elastin concentration, temperature of stock suspensions and time interval after the preparation of the stock suspension were investigated. The effect of different experimental parameters on elastin structure was investigated using atomic force microscopy.

AFM is a powerful imaging and characterization technique that enables the visualization and analysis of materials at the nano scale [14,15]. Unlike traditional optical microscopes that use light to observe samples, AFM operates through scanning a sharp probe over the surface of a material to measure the interactions between the probe and the surface [14,15]. The acquisition of high-resolution images and the precise measurements of various physical properties, such as topography [16], roughness [16] and mechanical stiffness [17,18], is carried out with atomic force microscopy (AFM) at sub-molecular resolution. AFM has proven to be a versatile tool for studying a wide range of materials, from biological molecules [19,20,21] and cells [22,23] to advanced materials and surfaces [24] and has played a significant role in advancing research in fields such as nanotechnology, materials science and biophysics.

## 2. Materials and Methods

### 2.1. Preparing Elastin Samples

#### 2.1.1. Preparation of Stock Suspensions

Elastin from bovine neck ligament (E1625, Sigma-Aldrich, Merck KGaA, Darmstadt, Germany) was dissolved in three different solvents, including Dulbecco’s phosphate-buffered saline (PBS) Gibco 14190, acetic acid (0.25 M) and ultrapure water, in three different concentrations (0.5% *w*/*v*, 1% *w*/*v*, 2% *w*/*v*), stored at four different temperatures (5 °C, 10 °C, 20 °C, 37 °C) and measured at different times (1 day, 1 week, 2 weeks, 1 month, 6 months) after the preparation of each stock suspension.

All the experiments were carried out during the same time period including all possible combinations of the different parameters. At each experiment, one parameter was changed and the other three were kept stable.

#### 2.1.2. Elastin Sample Preparation

Part of each elastin suspension (10–20 μL) was flushed on the substrate consisting of fresh cleaved mica discs (Product No: 50, Pelco, 9.9 mm diameter, Ted Pella, Inc., Redding, CA, USA). Subsequently, a washing step with ultrapure water was performed and samples were then air-dried in ambient conditions (room temperature) and used for AFM imaging. The same procedure was repeated for each experiment.

### 2.2. Atomic Force Microscopy

#### 2.2.1. AFM Imaging

AFM images of the elastin fibers were obtained in the air using a commercial microscope (Veeco Instruments, model: CP II). All the images were obtained at room temperature in a tapping mode at a scanning rate of 1 Hz, using an anisotropic AFM probe (MPP-11123-10, Βruker, Innova, Camarillo, CA, USA) having a spring constant of 40N /m and a tip radius of 8nm, operating at a resonance frequency of 300kHz. The image processing and the quantitative measurements, including the average values of fibers dimensions (diameters and heights), were made using the image analysis software (Digital Instruments-Scanning Probe Microscopy (DI-SPM) Lab ver.60.2, Veeco). Five images from each sample were taken from several locations (512 × 512 points per image) and with different image sizes (from 50 × 50 μm to 200 × 200 nm).

The samples were mounted directly on AFM metal specimen discs (12 mm diameter, Product No.16208, Ted Pella, Inc. Redding, CA, USA) with adhesive carbon tabs (12 mm diameter, model: G3347N, Agar Scientific, Essex, United Kingdom) in order to be more stable. Under each substrate, a copper finder grid (G2761C, 200 mesh, F1, Agar Scientific, Essex, United Kingdom) was glued in order to map the film surface and to ensure that the same area was sampled when multiple images were demanded [25,26,27,28]. The topographic AFM images are presented in a two-dimensional (2D) or three-dimensional (3D) color scale.

#### 2.2.2. Image Processing

The image processing and the quantitative measurements were made using the image analysis software that accompanied the AFM system DI SPM Lab ver.60.2, IP-image processing and data analysis ver.2.1.15 (Veeco) and the freeware scanning probe microscopy software WSxM 5.0 dev.9.1 [29]. The AFM quantitative measurements include a number of parameters such as height, profile, plot and distance measurements.

#### 2.2.3. Tip Geometry

AFM images are always affected by artifacts arising from tip convolution effects [30,31]. In this paper, the Canet-Ferrer et al. approach was used to minimize the effects of such errors in the quantitative analysis of the results [31]. The resulting errors can be significant for the case of nanofibrils. For the case that the tip radius is bigger than the effective height of the sample (i.e., $r_{tip}>h_{eff}$), the Canet-Ferrer et al. model is provided below:

(1) $$\frac{1}{2}w_{exp}=\Delta +\frac{1}{2}w_{eff}$$

and,

(2) $$\Delta =r_{tip}\cos\left[\arcsin\left(\left(r_{tip}-h_{eff}\right)/r_{tip}\right)\right]$$

In Equation (1), $w_{exp}$ and $w_{eff}$ are the expected and the effective width of the sample, respectively [31]. In addition, if $r_{tip}<h_{eff}$,

(3) $$\frac{1}{2}w_{exp}=r_{tip}+\Delta +\frac{1}{2}w_{eff}$$

and

(4) $$\Delta =(h_{eff}-r_{tip})tan\gamma$$

In Equation (4), γ is the tip to face angle [31].

## 3. Results

### 3.1. Elastin in Different Solvents

Elastin was suspended in ultrapure water at concentrations of 0.5% *w*/*v*, 1% *w*/*v* and 2% *w*/*v*. The resulting stock suspensions were stored at temperatures of 5 °C, 10 °C, 20 °C and 37 °C. After a time period of 1 day, 1 week and 1 month, 10–20 μL from each elastin suspension was flushed over the mica substrate discs. The samples were then air-dried and ready for imaging via AFM. The same procedure was repeated for acetic acid (0.25 M) and PBS medium. For each concentration/temperature and time interval, five images were obtained. In terms of measuring the diameters and heights of fibers, fibrils and nanofibrils, ten measurements were conducted for each fiber, fibril or nanofibril.

**Figure 1:**
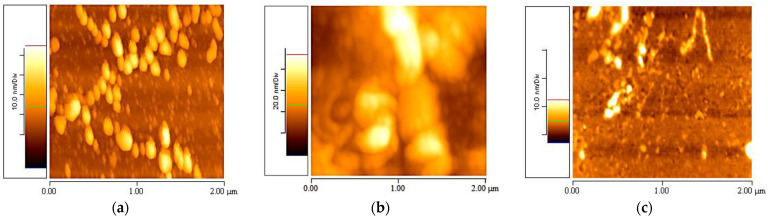
2 × 2 μm AFM topographic images taken one day after developing the stock elastin suspension with a concentration of 1% *w*/*v* at a temperature of 20 °C: (**a**) PBS, (**b**) Acetic acid, (**c**) Ultrapure water.

The initial experiments to investigate the appropriate suspension medium for elastin were conducted one day after preparing the stock suspension. The AFM images revealed the presence of globular coacervations (*z*-axis scale: 0–60 nm) in the elastin–PBS suspensions (Figure 1a), large and amorphous aggregations (*z*-axis scale: 0–100 nm) in the elastin–acetic acid suspensions (Figure 1b) and linearly formed coacervations (*z*-axis scale: 0–15 nm) in the elastin/ultrapure water suspension (Figure 1c). All the experiments were performed using various combinations of different parameters such as solvent, elastin concentration, temperature and time. Due to the large number of images obtained, only the most representative images from the three suspensions are presented.

Figure 1 presents 2 × 2 μm AFM topographic images taken on the first day after developing the stock elastin in water with a concentration of 1% *w*/*v* at 20 °C. It was observed that the elastin/ultrapure water suspension had fewer aggregations compared to the suspensions in PBS and acetic acid.

All experiments were conducted for each of the three suspensions, and it was observed that the most promising results for creating elastin fibrils were obtained in ultrapure water. Therefore, the following results are presented for the elastin–ultrapure water suspension.

### 3.2. Elastin Observed at Different Concentrations

The optimal concentration for elastin in the ultrapure water suspension was determined. Specifically, suspensions with three different elastin concentrations (0.5% *w*/*v*, 1% *w*/*v*, 2% *w*/*v*) in water were stored at various temperatures and observed at different time intervals.

**Figure 2:**
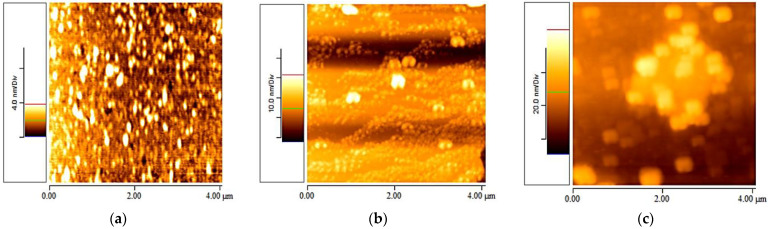
AFM topographic images taken one week after developing the stock elastin/ultrapure water suspension at a temperature of 20 °C are shown: (**a**) 0.5% *w*/*v* (4 × 4 μm), (**b**) 1% *w*/*v* (4 × 4 μm), (**c**) 2% *w*/*v* (4 × 4 μm).

The AFM figures depict the imaging of suspensions with different concentrations of elastin/water at 20 °C. As observed from the three AFM images, elastin nanofibrils were not formed even one week after the preparation of the stock suspensions. In the samples with elastin/ultrapure water 0.5% *w*/*v* (Figure 2a), a few low-height (*z*-axis scale: 0–4 nm) aggregations were observed. The suspension of elastin/ultrapure water 1% *w*/*v* (Figure 2b) exhibited numerous thin and long coacervations (*z*-axis scale: 0–20 nm). On the other hand, the samples with elastin/ultrapure water 2% *w*/*v* (Figure 2c) displayed very large aggregations (*z*-axis scale: 0–80 nm).

Figure 2 presents 4 × 4 μm AFM topographic images taken one week after developing the stock elastin/ultrapure water suspension at a temperature of 20 °C. It was observed that the elastin/ultrapure water suspension with a concentration of 1% *w*/*v* exhibited thin and long coacervations (unlike the globular ones observed in the other cases, i.e., concentrations of 0.5% *w*/*v* and 2% *w*/*v*). Therefore, it demonstrated suitable potential for creating elastin nanofibrils. Therefore, only the AFM images obtained from the elastin/ultrapure water suspension with a concentration of 1% *w*/*v* are presented in the following experiments.

### 3.3. Elastin Observed at Different Temperatures

In this section, the effect of temperature on the self-assembly of elastin in 1% *w*/*v* aqueous suspensions is investigated. The experiments were conducted at various combinations of temperature and time

**Figure 3:**
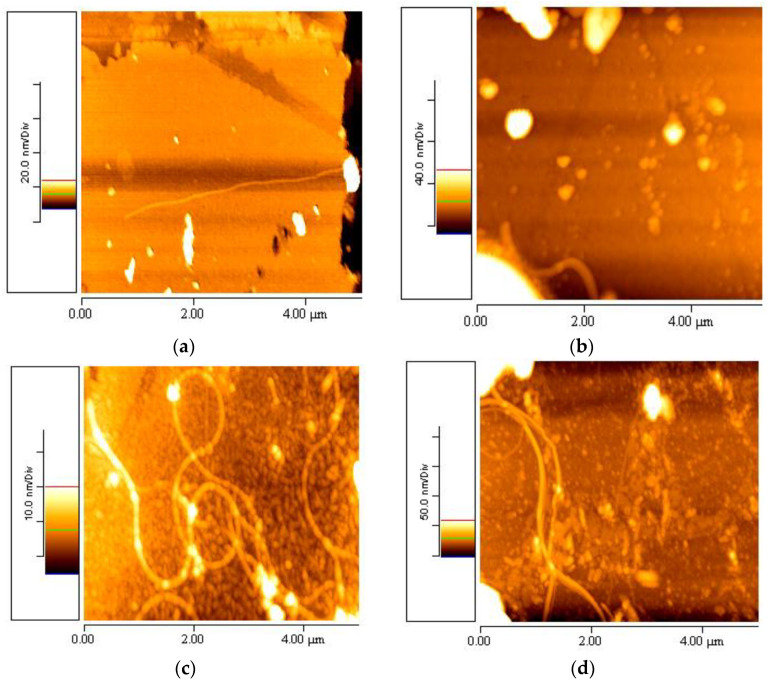
5 × 5 μm AFM topographic images taken one month after developing the stock 1% *w*/*v* elastin/ultrapure water suspension at the following temperatures: (**a**) 5 °C, (**b**) 10 °C, (**c**) 20 °C, (**d**) 37 °C.

Specifically, the aforementioned suspensions were stored at four different temperatures: 5 °C, 10 °C, 20 °C and 37 °C, and observed at various time periods. The AFM topographic images revealed that at 5 °C (Figure 3a), 10 °C (Figure 3b) and 37 °C (Figure 3d), there were only a few aggregations and one or two nanofibrils present in the samples of 1% *w*/*v* elastin/water. However, at 20 °C (Figure 3c), the first complete elastin mesh with fully formed nanofibrils was observed in the 1% *w*/*v* aqueous elastin stock suspension, with a *z*-axis scale of 0–25 nm.

Figure 3 presents representative 5 × 5 μm AFM topographic images from the stock of elastin/ultrapure water suspension with a concentration of 1% *w*/*v*, taken after one month of its preparation. It was observed that the 1% *w*/*v* elastin/ultrapure water suspension at 20 °C exhibited elastin nanofibrils with only a few aggregations. On the other hand, nanofibrils were not formed at 5 °C and 10 °C. For the case of 37 °C, one month after the creation of the stock solution, two to four nanofibrils were observed. The same number of fibers was observed from two months after the creation of the stock solution and onwards. No further increase in the number of fibers was determined for longer time intervals. It is worth noting that elastin consists of hydrophobic regions interspersed with cross-linking domains. At specific temperatures, such as those around physiological conditions, the hydrophobic regions of elastin interact with each other, resulting in protein aggregation and coacervation. These aggregations were more prominent at temperatures of 5 °C, 10 °C and 37 °C, as depicted in Figure 3a,b,d, respectively.

### 3.4. Elastin Observed at Different Periods of Time

**Figure 4:**
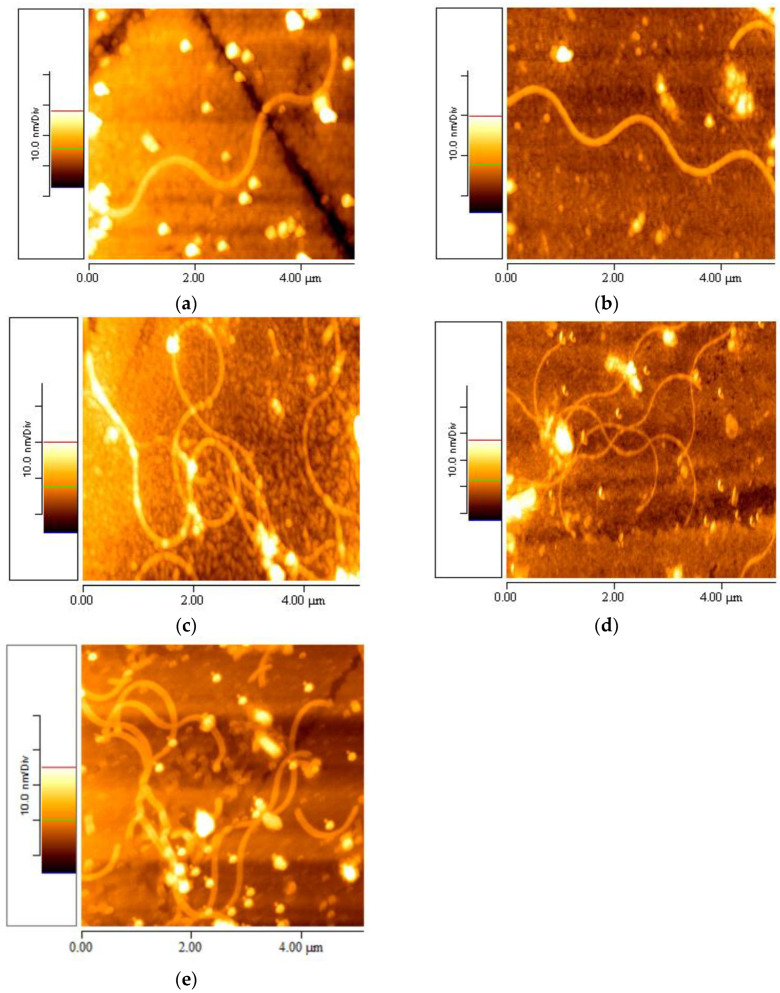
5 × 5 μm AFM topographic images taken after (**a**) two weeks, (**b**) three weeks, (**c**) one month, (**d**) four months and (**e**) six months from the preparation of the stock suspension of elastin in ultrapure water with a concentration of 1% *w*/*v*, stored at 20 °C.

**Figure 5:**
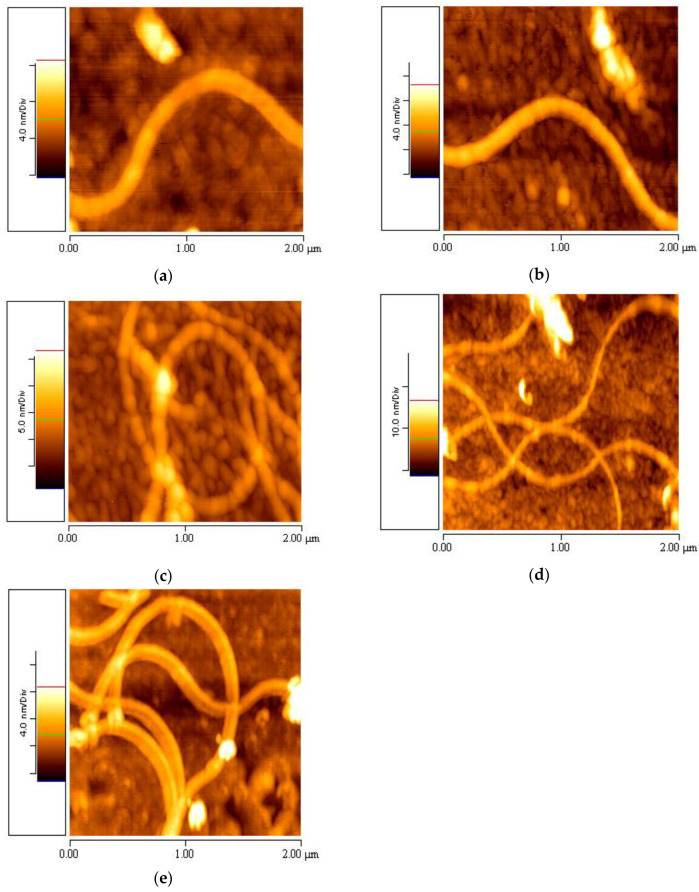
2 × 2 μm AFM topographic images taken after (**a**) two weeks, (**b**) three weeks, (**c**) one month, (**d**) four months and (**e**) six months from the preparation of the stock suspension of elastin in ultrapure water with a concentration of 1% *w*/*v*, stored at 20 °C.

The stock aqueous elastin suspension with a concentration of 1% *w*/*v* was stored at 20 °C and measured and observed via AFM at different time intervals: one day, two weeks, one month and six months. The AFM images reveal that one week after the preparation of the 1% *w*/*v* aqueous elastin stock suspension at 20 °C, no formation of elastin fibrils was observed, but numerous aggregations were present (Figure 1c and Figure 2b). The first elastin nanofibrils were observed two weeks after the development of the stock suspension (Figure 4a and Figure 5a), and as time progressed, reaching one month, the first fully formed elastin mesh of nanofibrils was observed (Figure 4c and Figure 5c). This dense mesh of fibrils continued to extend over time, as observed in the AFM images taken six months later (Figure 4d,e and Figure 5d,e).

After six months, differences in the number of fibrils were observed. As a result, the increasing formation of nanofibrils over time demonstrated qualitatively in this research.

A statistical study was also conducted to determine the increase in the number of fibrils formed under different conditions. Specifically, AFM images with a size of 5 × 5 μm (using the sample with a 0.1% *w*/*v* elastin-ultrapure water solution at 20 °C) were tested at various time intervals (five images per time interval). After two weeks, one or two individual nanofibrils of finite length were observed in each image. After one month, five to seven very long nanofibrils tangled together were recorded. From two months to six months, approximately fifteen entangled and long nanofibrils were observed. Additionally, at two months, the self-assembly from nanofibril to fibril and then to fiber was observed for the first time and thoroughly investigated.

In addition, a statistical study was conducted to quantify the nanofibrils using AFM images (5 μm × 5 μm) after the creation of the 0.1% *w*/*v* elastin-ultrapure water solution at 37 °C over time. For each time interval, five images were analyzed. After one month from the creation of the stock solution, two to four nanofibrils were observed. The same number of fibers was observed from two months after the creation of the stock solution onwards.

Additional experiments were conducted using the 0.1% *w*/*v* elastin–ultrapure water solution at 5 °C and 10 °C, with different solvents (PBS, acetic acid) and with elastin concentrations of 0.5% *w*/*v* and 2% *w*/*v*. These experiments were carried out for a period of up to 3 months from the creation of the stock solution. In these cases, no fibrils were observed.

This was the reason why the experiments were focused both qualitatively and quantitatively on the 0.1% *w*/*v* elastin–ultrapure water solution at 20 °C.

### 3.5. Geometrical Characteristics of Elastin Nanofibrils 

**Figure 6:**
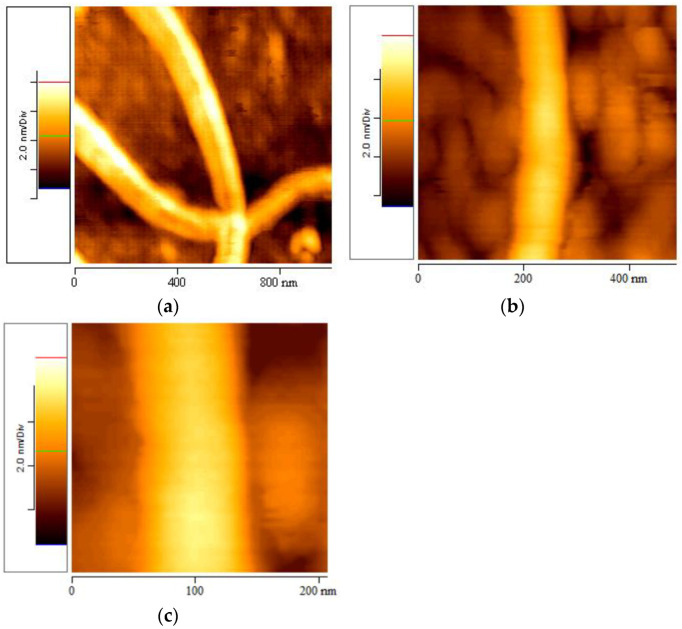
AFM topographic images, obtained at different resolutions, after six months from the preparation of the stock suspension of elastin in ultrapure water at 1% *w*/*v* concentration, stored at 20 °C: (**a**) 1 × 1 μm, (**b**) 500 × 500 nm, (**c**) 200 × 200 nm.

**Figure 7:**
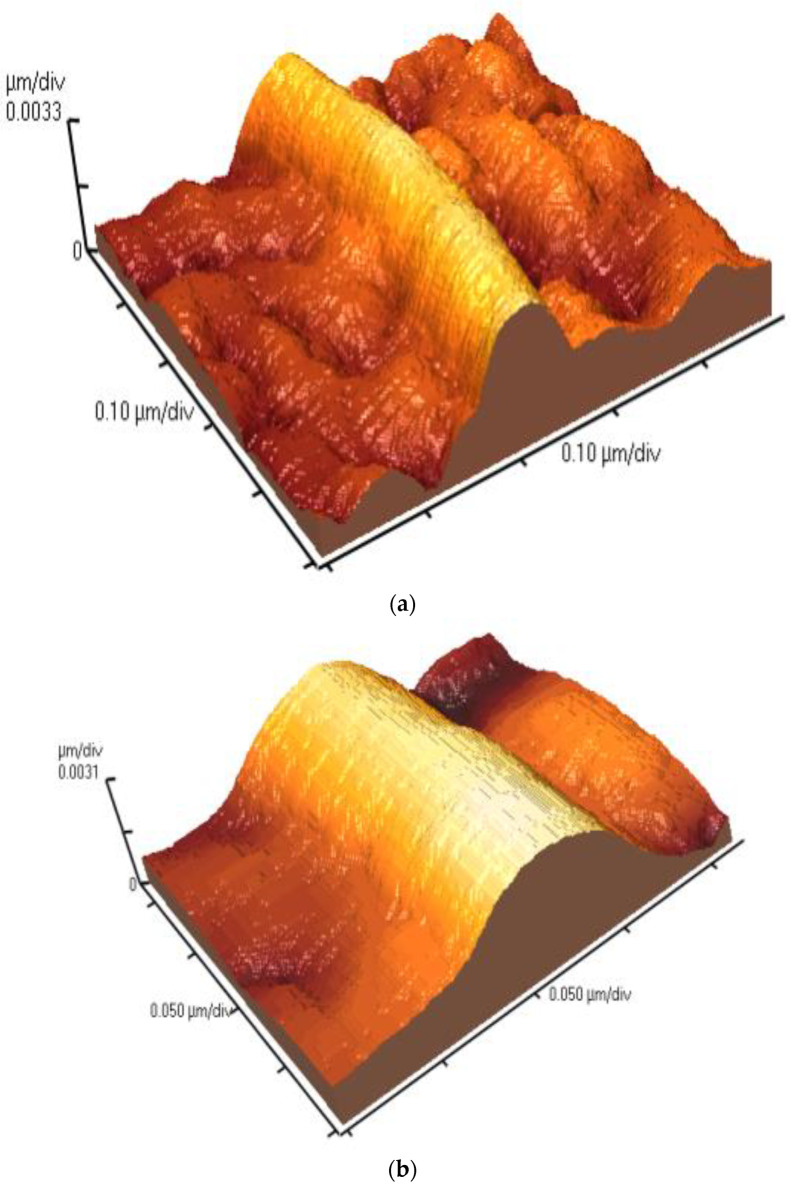
3D AFM topographic images, obtained at different resolutions, after six months from the preparation of the stock suspension of elastin in ultrapure water at 1% *w*/*v* concentration, stored at 20 °C: (**a**) 500 × 500 × 6.6 nm, (**b**) 200 × 200 × 6.2 nm.

AFM topographic 2D images (Figure 6) and corresponding 3D images (Figure 7) of the elastin samples in ultrapure water at 1% *w*/*v* concentration stored at 20 °C were obtained at higher magnifications ranging from 2 × 2 μm to 0.5 × 0.5 μm. These images, obtained six months after the preparation of the stock suspension, further confirmed the presence of elastin nanofibrils.

The geometrical characteristics of the nanofibrils were measured using the appropriate AFM software program. The average values of the diameters and heights of the elastin nanofibrils were (134.5 ± 23.3) nm and (4.5 ± 0.7) nm, respectively. 

However, it is important to consider the errors resulting from the tip geometry. The tip radius used in all cases was approximately 8 nm. Assuming an effective height of 4 nm for the nanofibrils, Equation (2) yields Δ ≈ 7 nm. In addition, according to Equation (1),

$$w_{exp}-w_{eff}=2\Delta \approx 14\;\mathrm{nm}$$

Thus, the convolution error reduction is $1.75r_{tip}$ as expected in [31]. Thus, for the case of nanofibrils, the error is approximately 10%. Therefore, the real diameters of the nanofibrils are smaller and equal to (121±21) nm. 

The error for the case of fibers and fibrils is even smaller. In particular, through considering approximately $\gamma =15^{{}^{\circ}}$, and $h_{eff}\approx 25\;\mathrm{nm}$ for the case of fibrils, according to Equation (4), Δ≈4.6 nm and according to Equation (3):

$$w_{exp}-w_{eff}=2\left(\Delta +r_{tip}\right)\approx 25.2\;\mathrm{nm}$$

Thus, the error is approximately 7%. 

For the case of fibers, assuming $h_{eff}=80\;\mathrm{nm}$, Δ≈19.44 nm and

$$w_{exp}-w_{eff}=2\left(\Delta +r_{tip}\right)\approx 54\;\mathrm{nm}$$

the error is approximately 9%. Thus, the results should be revised. After accounting for the tip geometry corrections, the diameters of the fibrils are (321.3 ± 21.0) nm. Similarly, for the fibers, the corrected diameters are (549.64 ± 25.4) nm.

**Figure 8:**
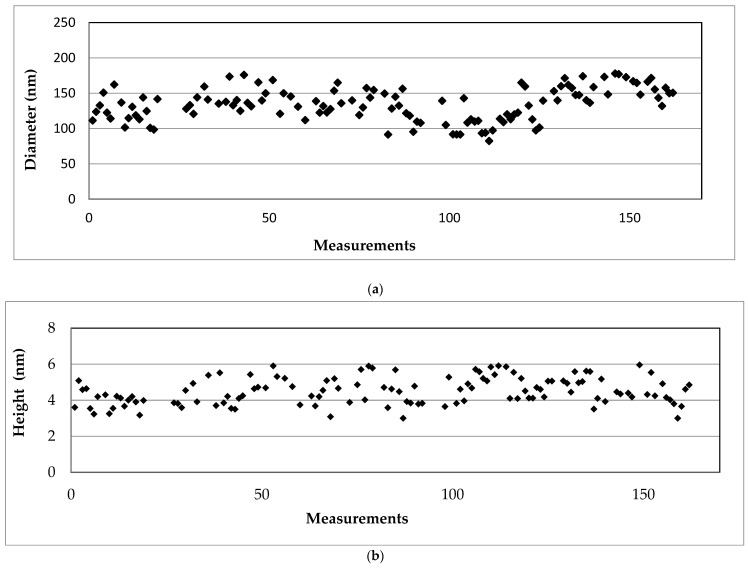

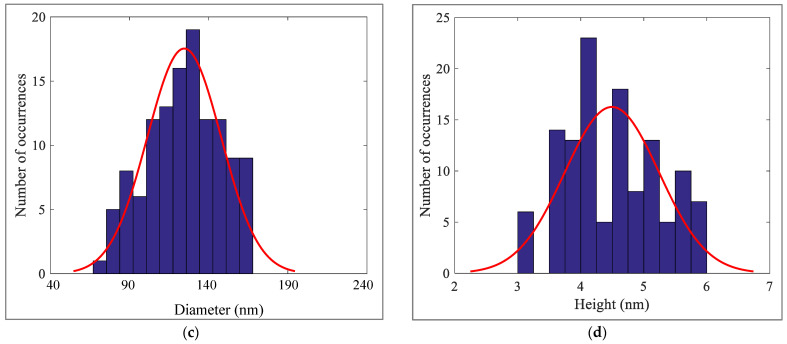
Measurements of the geometrical characteristics of 160 elastin nanofibrils: (**a**) diameter (nm), (**b**) height (nm). Histogram for the same measurements of (**c**) diameter values and (**d**) height values. The diameters, in terms of mean value ± standard deviation, resulted in 121 ± 21 nm. The heights, in terms of mean value ± standard deviation, resulted in 4.5 ± 0.7 nm.

Representative images are demonstrated for 160 nanofibrils (Figure 8a,b). It is important to note that the statistical analysis of protein size and shape was conducted using images such as Figure 5 and Figure 6, which had dimensions ranging from 2 × 2 μm to 0.5 × 0.5 μm.

In Figure 8c,d, histograms that present the diameters and the heights for 160 nanofibrils are shown. The data were fitted to Gaussian functions. For the case of fibril diameters,

(5) $$f\left(d\right)=\frac{1}{21\sqrt{2\pi }}e^{-\frac{1}{2}{\left(\frac{d-121}{21}\right)}^{2}}$$

In addition, for fibril heights,

(6) $$f\left(h\right)=\frac{1}{0.7\sqrt{2\pi }}e^{-\frac{1}{2}{\left(\frac{h-4.5}{0.7}\right)}^{2}}$$

It is interesting to investigate whether there is a pattern that can describe the geometric characteristics of the fibrils/fibers that are formed using the aforementioned protocol. Assuming an elliptical cross-sectional area of the fibril/fiber (where the cross-sectional area can be calculated as A=πRh, where R is the radius and h is the height of the fibril), it has been found that there is a linear relationship between the area and diameter. 

**Figure 9:**
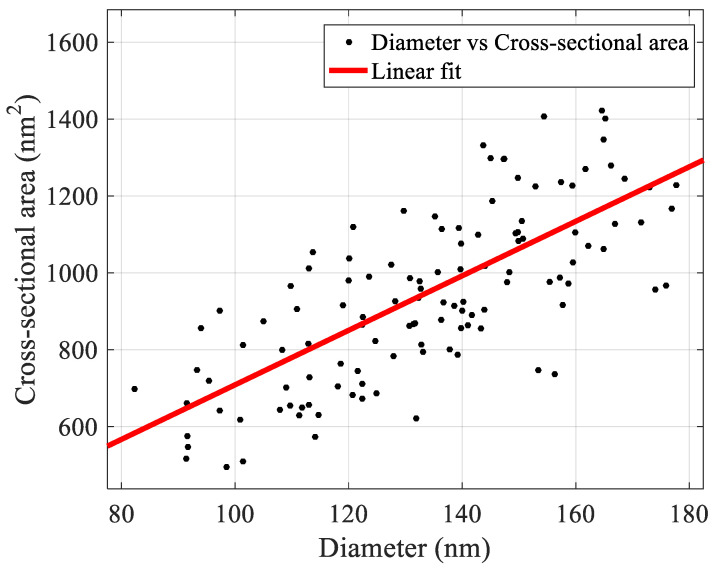
The cross-sectional area of the fibrils with respect to their diameters. The data were fitted to a linear curve A=7.088(nm)d.

For instance, it was observed that in the case of nanofibrils (based on the 160 measurements presented in Figure 7), the following equation can be applied (Figure 9):

$$A=7.088\left(\mathrm{nm}\right)d,\mathrm{where}A\;\mathrm{is}\;\mathrm{calculated}\;\mathrm{in}\;{\mathrm{nm}}^{2}$$

In conclusion, the formation of nanofibrils over different conditions including suspension medium, elastin concentration, temperature of stock suspensions and time interval after the preparation of the stock suspension were demonstrated qualitatively and quantitatively in this research.

### 3.6. Self-Assembly Imaging of Elastin

**Figure 10:**
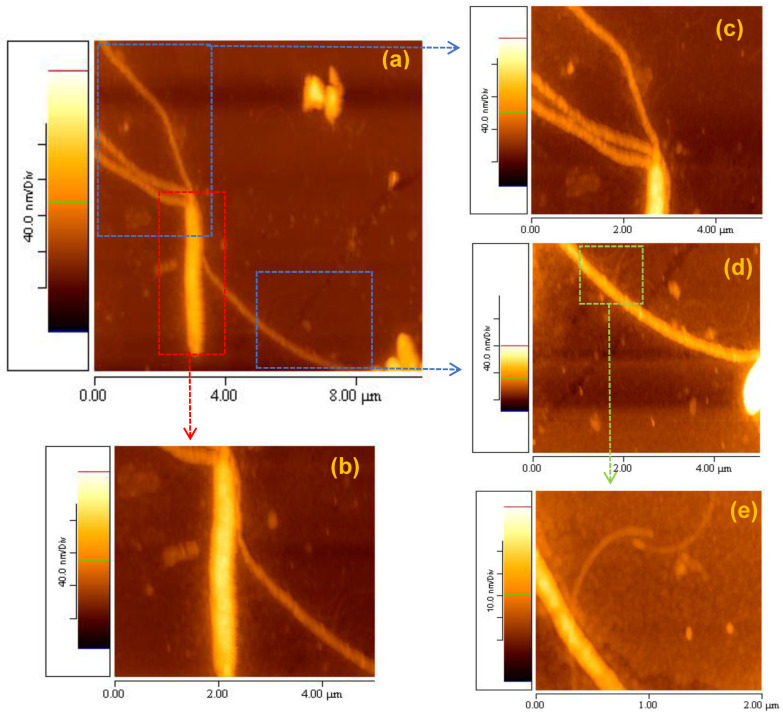
AFM topographic images of elastin self-assembly (**a**): (**b**) fiber → (**c_,_ d**) fibrils → (**e**) nanofibrils.

It was observed, even from the initial experiments, that the self-assembly process of elastin proceeded from nanofibrils to fibrils and eventually to fibers. The formation of the first nanofibrils required a minimum of two weeks after the preparation of the stock suspension of elastin in ultrapure water 1% *w*/*v* at 20 °C (Figure 10d). After almost two weeks, these nanofibrils underwent self-assembly to form fibrils (Figure 10c). Finally, approximately six weeks after the preparation of the stock aqueous suspension, elastin fibers were produced from the self-assembled fibrils (Figure 10b). Hence, in Figure 10a, an AFM topographic image of size 10 × 10 μm captures the presence of one elastin fiber and four fibrils (three located at the top left and one on the right side of the fiber). Although there were several faint nanofibrils, their observation was challenging due to the significant difference in height between the fibers (88.8 ± 5.7 nm) and the nanofibrils (4.5 ± 0.7 nm). Figure 10b presents a higher-magnification AFM topographic image (5 × 5 μm) of the same elastin fiber. 

In Figure 10c,d, two AFM topographic images (5 × 5 μm) showcase the four fibrils (c: three from the top left and d: one on the right side of the observed fiber), while Figure 10e displays an AFM topographic image (2 × 2 μm) of the nanofibrils, supporting the assumption of a self-assembly process.

**Figure 11:**
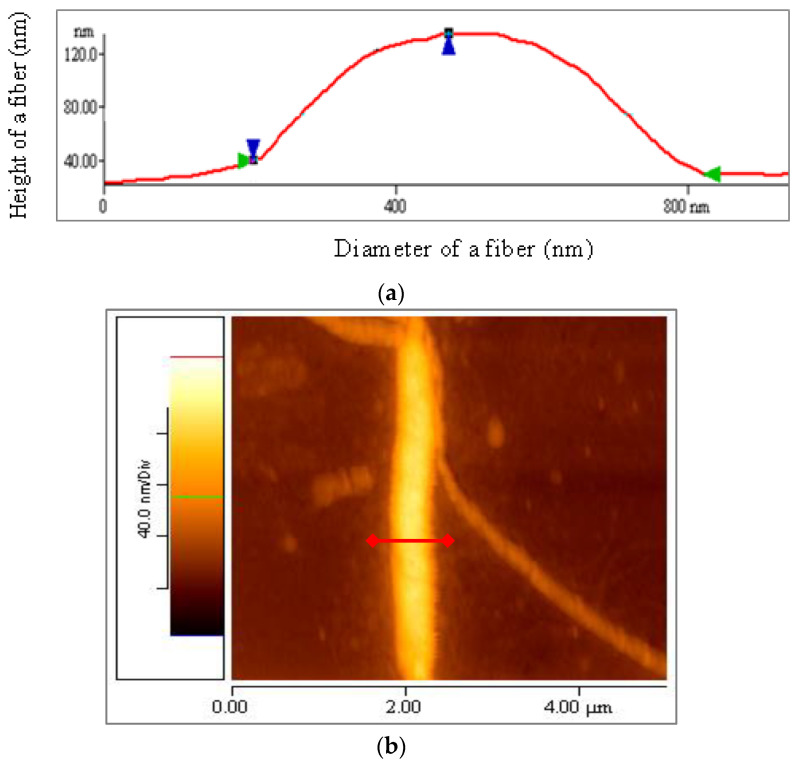
(**a**) Example of AFM measurement during data processing [the height of a fiber was measured through calculating the vertical distance between blue markers (blue triangles) and the diameter through the horizontal distance between green markers (green triangles)], (**b**) Indicative height and diameter profile plot of a fiber. [Red line of (**a**) match with red line of (**b**)].

In Figure 11a, an illustrative height and diameter profile plot of a fiber is displayed. The height of a fiber, fibril or nanofibril was determined through measuring the vertical distance between blue markers (blue triangles), while the diameter was measured through calculating the horizontal distance between green markers (green triangles). Corrections due to the tip geometry were also made, as already mentioned. In Figure 11b, a red line is highlighted as an example of an AFM measurement during the data processing of the aforementioned profile plot. The average values of diameters and heights for a significant number of elastin nanofibrils, fibrils and fibers are presented in Table 1.

## 4. Discussion

Numerous techniques have been described for obtaining fibers and fibrous scaffolds from elastin-based materials, including self-assembly processes and electrospinning [32]. Towards this direction, this paper focuses on the investigation of the self-assembly process of elastin fiber structures using AFM imaging, with various experimental parameters being studied, including suspension medium, elastin concentration, temperature of stock suspensions and time interval after preparation. Elastin is a promising biomaterial due to its low inflammatory response [33], and as it is a major protein found in connective tissue, this research prioritizes the investigation of its self-assembly procedure at the nano scale.

In a previous study, Srokowski and Woodhouse [34] used AFM 2D imaging to analyze the surface topography of ELP-coated surfaces in PBS buffer. They found no discernable difference in surface features between uncoated and ELP-coated surfaces, indicating that each of the ELPs adhered to the underlying substrate and formed a conformal coating. However, in this particular research, suspensions of elastin were prepared using PBS and spherical aggregations were observed. When ultra-pure water was used as a suspension medium instead, 3D fibrils and fibers of elastin were imaged.

In earlier research, Dandurand et al. [35] conducted a comparison of the thermal behavior of water in solutions containing different peptides from elastin, which either coacervate or form structures, in order to better understand the role of water in the mechanism of aggregation. Additionally, Koga et al. [36] created ELP segment layers on a hydrophobic glass surface which were then immersed in an aqueous solution at 37 °C, but no elastin fiber formation was observed. Similarly, in the present study, experiments carried out at temperatures of 5 °C, 10 °C and 37 °C did not reveal any elastin fibers. As a result, further investigations were conducted using the stock elastin/ultrapure water suspension at a temperature of 20 °C, where 3D elastin fibers were successfully imaged.

Pepe et al. [37] conducted previous research focused on investigating the morphology of formed aggregates, with S4 peptide samples without TBS solvent exhibiting the formation of long and flexible fibers. AFM images obtained from these samples only showed highly aggregated globular structures, indicating that the solvent had an inhibitory effect on the formation of the peptide. In a separate study by del Mercato et al. [38], synthetic peptide fibrils were characterized structurally via self-assembly. To generate these fibrils, the peptide was suspended in water and incubated in a vial tube for a period of several weeks, typically ranging from 3 to 5 weeks, to allow for the formation of mature fibrils. 

Bochicchio et al. [39] observed similar results not only with other elastin-related peptides but also in the present study, where the elastin suspension was stored for two weeks before 3D elastin fibrils could be imaged. However, this paper presents new and detailed insights into the structural and geometrical characteristics of elastin fibers, fibrils and nanofibrils. Therefore, the qualitative and quantitative measurements presented in this study serve as evidence for the process of elastin self-assembly. In addition, Cao et al. demonstrated how a temperature increase from 20 °C to 80 °C affects the self-assembly of ELPs [40]. Tarakanova et al. used a principal component analysis to consider the ensemble of structures accessible to tropoelastin at 37 °C, at which tropoelastin naturally self-assembles into aggregated coacervates [41]. Due to the challenging nature of studying the intrinsic properties of elastin and tropoelastin, the comprehension of crucial aspects of the assembly process is challenging [42]. Therefore, current research in this field is regarded as state-of-the-art [42].

In addition, an interesting work was presented by Bracalello et al. In particular, they focused on the preparation and structural characterization of nanofibers from a chimer-ic-polypeptide-containing resilin and elastin domain [43]. The purpose of their research was to enhance its cell-binding ability through introducing a specific fibronectin-derived Arg-Gly-Asp (RGD) sequence. In another recent work, Alvisi et al. investigated the self-assembly of elastin-like polypeptide brushes on silica surfaces and nanoparticles [44].

As already mentioned, elastin is a protein that provides elasticity and resilience to tissues in the body, such as the skin, lungs and blood vessels. Controlling elastin’s properties is important for various applications in the fields of tissue engineering, drug delivery and medicine [45,46]. For example, elastin is used in tissue engineering to create artificial tissues that have similar mechanical properties as natural tissues [47]. Elastin may be incorporated into skin substitutes to improve the elasticity of artificial skin. In addition, elastin-like polypeptides (ELPs) are a type of synthetic elastin that can be used as drug carriers. ELPs have the ability to self-assemble into nanoparticles, which can be loaded with drugs and targeted to specific tissues [48]. It should be also noted that elastin is an essential component of blood vessels and, therefore, it is used in the production of vascular grafts. These grafts are used to repair or replace damaged blood vessels in the body [49]. Furthermore, elastin plays a crucial role in the structural development of lungs, particularly during the alveolar stage. Research has established that the breakdown of elastin is a critical factor in the development of chronic obstructive pulmonary disease. Additionally, the inability of lung cells to repair damaged elastic fibers exacerbates the disease process, resulting in long-term degenerative illness and compromised lung function [50]. Due to their intricate molecular composition, large size and dependence on several assisting proteins for proper assembly, elastic fibers are considered one of the most challenging matrix structures to restore. Regarding wound healing, elastin-like polypeptides (ELPs) may be used as excellent materials for fabricating biocompatible scaffolds and other products for wound management [51]. These biomolecules exhibit solubility below critical temperatures and undergo aggregation at higher temperatures, making them a fascinating resource for designing diverse nanobiomaterials [45].

## 5. Conclusions

Biomedical applications, particularly tissue engineering, can derive significant benefits from elastin nanofibrous meshes. When constructing an engineered tissue, a biomaterial is first designed using various processing methods such as solvent casting and self-assembly. The biomaterials should exhibit behavior similar to that found in the body, enabling the tissues to achieve healing. As structural characteristics play a crucial role for most cells, the choice of biomaterial for scaffold design holds immense importance. These were some of the reasons why it was necessary to investigate the process of self-assembly of elastin from nanofibrils to fibrils and fibers under various experimental parameters. Imaging was performed using atomic force microscopy (AFM) due to its high resolution at the nano scale. It is also significant to note that appropriate corrections were made to account for the effects of tip geometry. The error resulting from the tip geometry was in the range of 7–10%, with the largest error observed in the case of nanofibrils. The findings indicated that the optimal conditions for observing the self-assembly process and the formation of elastin fibrils at the nanoscale were a 1% *w*/*v* concentration of elastin in ultra-pure water at a temperature of 20 °C, with a minimum incubation period of two weeks after the preparation of the stock suspension.

In conclusion, the above research can be a step forward towards a better understanding of elastin self-assembly under various parameters. These results are anticipated to offer viable solutions for the fabrication of protein-inspired nanostructures that possess specific physical and chemical properties, catering to the needs of various applications in biotechnology and tissue engineering. 

## Figures and Tables

**Table 1:** Average values of diameter and height of fiber, fibril and nanofibril.

	Diameter (nm) (Average Value ± Standard Deviation)	Height (nm) (Average Value ± Standard Deviation)
Fiber	549.64±25.4	88.8±5.7
Fibril	321.3±21.0	27.7±3.3
Nanofibril	121±21.0	4.5±0.7

## Data Availability

Not applicable.
